# Resting-state functional magnetic resonance imaging of high altitude patients with obstructive sleep apnoea hypopnoea syndrome

**DOI:** 10.1038/s41598-020-72339-2

**Published:** 2020-09-23

**Authors:** Zongyuan Qin, Dongjie Kang, Xiang Feng, Demin Kong, Fangfang Wang, Haihua Bao

**Affiliations:** Department of Medical Imaging Center, Affiliated Hospital of Qinghai University, Xining, 810001 China

**Keywords:** Respiratory distress syndrome, Clinical trial design

## Abstract

The objective of the study was to observe brain function changes in Obstructive Sleep Apnoea Hypopnoea Syndrome (OSAHS) patients at high altitude. Resting-state functional magnetic resonance imaging (rs-fMRI) in patients with OSAHS was assessed using regional homogeneity (ReHo), amplitude of low frequency fluctuation (ALFF) and functional connectivity (FC). In this study, 36 male patients with OSAHS and 38 healthy male subjects were recruited from high-altitude areas, specifically, altitudes of 2,000–3,000 m. OSAHS was diagnosed by polysomnography (PSG). The blood oxygen level-dependent (BOLD) signals of OSAHS patients and healthy controls in the resting state were obtained and compared using ReHo, ALFF and FC methods. The posterior cingulate cortex (PCC) was selected as the seed region in the comparison of FC between the two groups. Compared with the healthy control group, multiple brain functions in the OSAHS patient group were different. There were correlations between the brain function values of some brain regions and demographic data. We also found that in contrast to earlier findings with individuals in plains areas, the brain function at the frontal lobe and the precuneus were higher in OSAHS patients, and the PCC showed higher FC with the left caudate, which may be due to the high-altitude hypoxic environment.

## Introduction

Obstructive sleep apnoea-hypopnoea syndrome (OSAHS) is a sleep-related breathing disorder characterized by frequent upper airway collapse during sleep and sleep snoring accompanied by superficial pauses in breathing. Hypoxia is one of the most influential factors affecting the function of the body. High-altitude areas have a special geographical environment, involving hypoxia, cold temperatures and high diurnal temperature variation. Hypoxia is the most likely to damage the structure and function of the brain, and it is one of the most characteristic features of high-altitude environments and one of the biggest influences on human health. Therefore, it is of great significance to understand changes in brain function in OSAHS patients in the plateau area.

Previously, researchers have used voxel-based analysis (VBM) and diffusion tensor imaging (DTI) methods to explore the changes in brain structure and microstructure in patients with OSAHS and used the ReHo technique to investigate changes in brain function and brain networks in patients with OSAHS^1–11^. However, there are no data on the study of resting-state functional magnetic resonance imaging (rs-fMRI) in OSAHS patients at high altitude.

Blood oxygen level-dependent (BOLD) signals have been correlated with cerebral blood volume (CBV), cerebral blood flow (CBF) and cerebral metabolic rate of oxygen (CMRO_2_). The blood contains a large amount of oxygenated haemoglobin, and deoxyhaemoglobin (paramagnetic) is converted from oxygenated haemoglobin (diamagnetic). The combination of the blood magnetic contributions in activated regions lead to a reduction of T2* relaxation time and a decreased MR signal^12^. This signal difference, named the BOLD signal^12^, can be picked up and exploited to infer neuronal activation via a vascular phenomenon (neuro-vascular coupling). As a result, following cerebral activations or at rest, when the CBV and CBF and CMRO_2_ of local brain regions vary, the amount of deoxyhemoglobin also changed, which links the BOLD signal to brain functions. Rs-fMRI uses the spontaneous BOLD signal fluctuations acquired in the absence of a stimulus or a task, and can be exploited by measuring changes in Regional homogeneity (ReHo), amplitude of low frequency fluctuation (ALFF) and functional connectivity (FC) values. Such resting state brain function measures "default mode" brain functioning. Thus, the default mode network is a group of brain regions showing higher levels of activity when subjects are awake and not involved in any specific mental exercise.

ReHo and ALFF were originally proposed by Zang et al.^13,14^. The functional interaction between different areas of the brain is called FC^15^. Changes in ReHo, ALFF, and FC values can be used to reflect spontaneous changes in brain metabolism, brain function, and neural activity. Therefore, it is of profound significance to use rs-fMRI technology like ReHo, ALFF and FC measurements to conduct in-depth research on OSAHS patients and explore changes in their brain at high altitude. Indeed, hypoxia caused by OSAHS and increased by high altitude could be linked to vascular, metabolic and perhaps anatomic (brain connections) changes and may, at a long time scale, affect brain oxygenation and consequently able to modify resting state functions and connectivity.

Although these changes are inter-related and complex, the aim of this study is to investigate and describe the changes in BOLD-related brain activity occurring in OSAHS patients compared to controls, both at high altitude.

## Results

### Subjects

Demographic and physiological data of the OSAHS group and control group, including altitude, age, years of education, body mass index (BMI), apnoea-hypopnoea index (AHI) and mean saturation of blood oxygen (MSaO_2_) are presented in Table 1.

**Table 1:** Demographic and physiological data.

	Patients with OSAHS (n = 36)	Healthy controls (n = 38)	T	P
	Mean ± standard deviation	Mean ± standard deviation		
Altitude (m)	2,422.89 ± 136.28	2,389.32 ± 149.46	1.008	0.317
Age (years)	48.50 ± 7.15	46.13 ± 7.02	1.438	0.155
Education (years)	8.89 ± 3.40	8.53 ± 2.33	0.532	0.597
BMI (kg/m^2^)	29.04 ± 2.76	23.41 ± 2.45	9.299	< 0.001
Apnoea–hypopnoea index (AHI)	58.78 ± 21.39	3.04 ± 1.13	15.610	< 0.001
MSaO_2_ (%)	82.92 ± 4.17	91.73 ± 1.54	11.945	< 0.001

*MSaO*_*2*_ mean saturation of blood oxygen.

### ReHo

**Figure 1:**
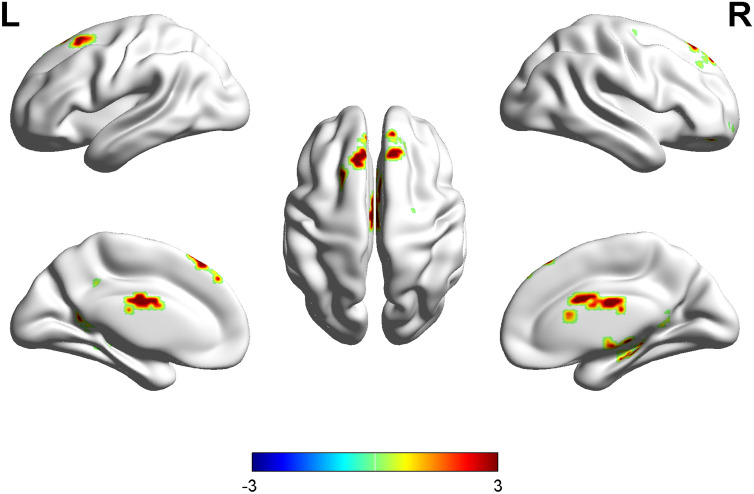
Maps of ReHo changes in OSAHS patients compared with the controls. Areas in red are regions where the ReHo value was significantly higher. *L* left, *R* right.

Compared with the healthy control group, the OSAHS patient group had higher ReHo values in the left superior frontal gyrus, right anterior cingulate, left parahippocampus, right postcentral gyrus, right hippocampus and right precuneus. Compared with the healthy control group, the OSAHS patient group had decreased ReHo values in the left cuneus and the left precuneus. A cluster was defined as a block of continuously connected voxels containing more than 22 voxels as the threshold value (Table 2 and Fig. 1).

**Table 2:** Brain regions with significant differences in ReHo values between the OSAHS patients and healthy controls.

Brain regions	Voxels	Peak MNI coordinates			Peak *t*-score
		X	Y	Z	
**OSAHS > control**					
Superior frontal gyrus_L	20	− 12	28	54	4.75
Anterior cingulate_R	8	3	12	27	3.69
Parahippocampus_L	21	− 17	− 22	− 23	4.85
Postcentral gyrus_R	7	29	− 32	38	4.15
Hippocampus_R	47	32	− 36	1	4.56
Precuneus_R	17	11	− 49	19	4.35
**OSAHS < control**					
Cuneus_L	9	− 3	− 81	39	− 4.32
Precuneus_L	10	− 2	− 78	40	− 4.49

P < 0.001 (cluster size > 22 voxels, AlphaSim correction, threshold = 3.43).

### ALFF

**Figure 2:**
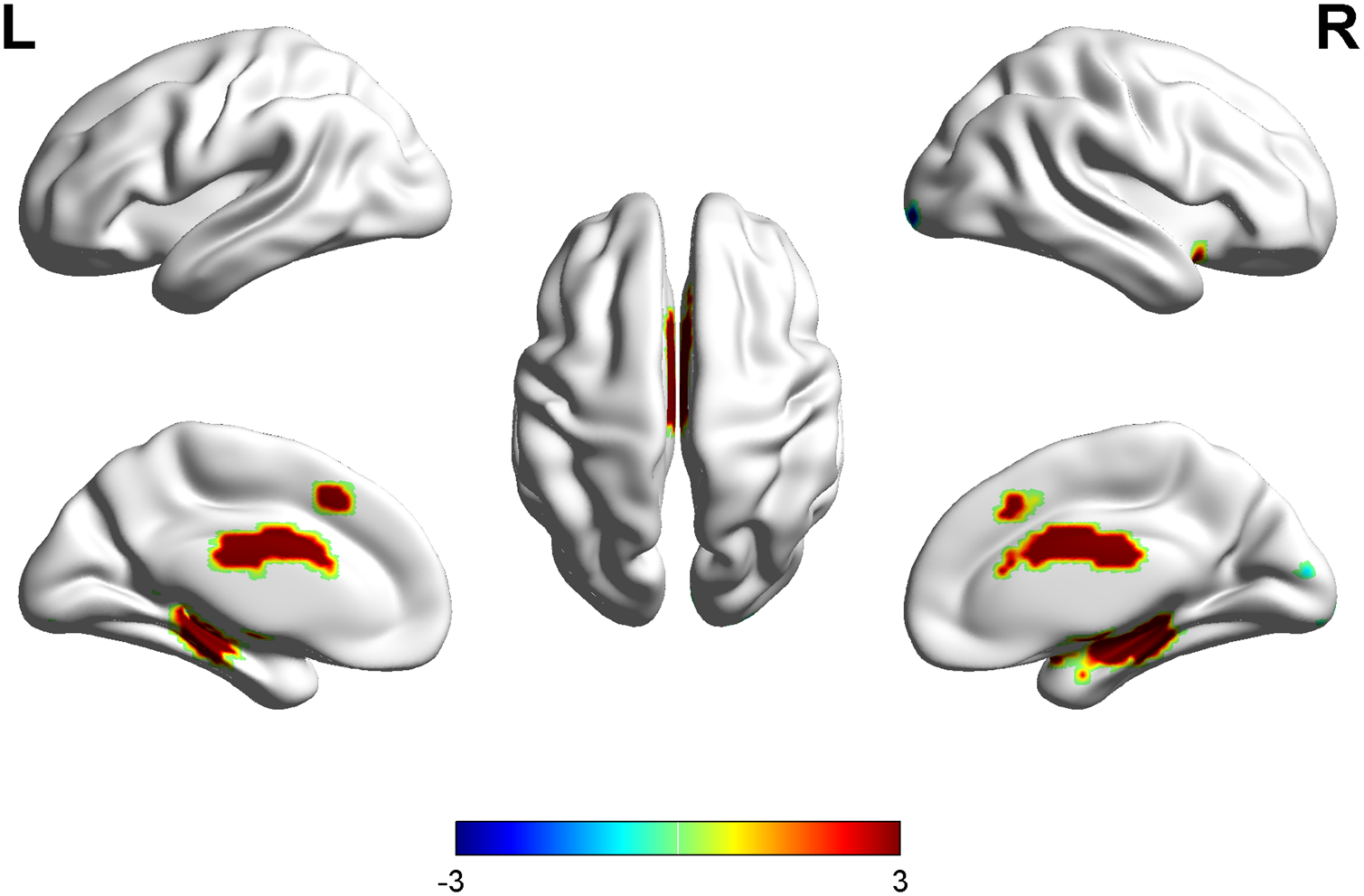
Maps of ALFF changes in the OSAHS patients compared with the controls. Areas in red are regions where the ALFF value was significantly higher. *L* left, *R* right.

Compared with the healthy control group, the OSAHS patient group had higher ALFF values in the right middle cingulate, left medial superior frontal gyrus, right anterior cingulate, right hippocampus and left parahippocampus. Compared with the healthy control group, the OSAHS patient group had decreased ALFF values in the right calcarine, right inferior occipital gyrus, right middle occipital gyrus, left calcarine and right cerebellum_7b. A cluster was defined as a block of continuously connected voxels containing more than 22 voxels as the threshold value (Table 3 and Fig. 2).

**Table 3:** Brain regions with significant differences in ALFF values between the OSAHS patients and healthy controls.

Brain regions	Voxels	Peak MNI coordinates			Peak *t*-score
		X	Y	Z	
**OSAHS > control**					
Middle cingulate_R	11	12	21	39	4.71
Medial superior frontal gyrus_L	20	− 5	22	42	4.44
Anterior cingulate_R	27	7	11	23	5.39
Hippocampus_R	158	26	− 14	− 14	6.49
Parahippocampus_L	82	− 19	− 20	− 24	5.15
**OSAHS < control**					
Calcarine_R	13	18	− 102	1	− 4.04
Inferior occipital gyrus_R	12	28	− 99	− 2	− 4.29
Middle occipital gyrus_R	8	27	− 96	0	− 4.08
Calcarine_L	16	− 3	− 89	− 9	− 3.89
Cerebelum_7b_R	7	42	− 69	− 56	− 4.19

P < 0.001 (cluster size > 22 voxels, AlphaSim correction, threshold = 3.43).

### FC

**Figure 3:**
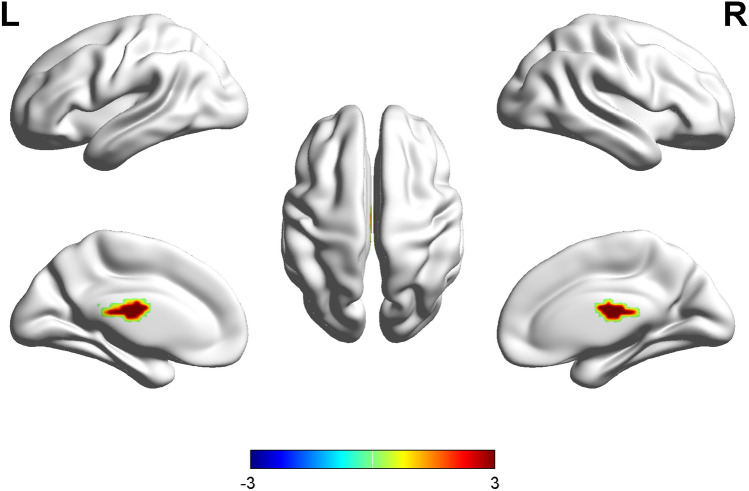
Maps of FC changes in the OSAHS patients compared with the controls. Areas in red are regions where functional connectivity with the PCC was significantly higher. *L* left, *R* right.

In the OSAHS patients, the PCC showed higher FC with the left caudate and left thalamus. The patients had no regions where functional connectivity with the PCC was significantly decreased. A cluster was defined as a block of continuously connected voxels containing more than 22 voxels as the threshold value (Table 4 and Fig. 3).

**Table 4:** Brain regions with significant differences in FC with PCC between the OSAHS patients and healthy controls.

Brain regions	Voxels	Peak MNI coordinates			Peak *t*-score
		X	Y	Z	
**OSAHS > control**					
Caudate_L	31	− 17	− 5	22	3.92
Thalamus_L	47	− 8	− 15	19	4.41

P < 0.001 (cluster size > 22 voxels, AlphaSim correction, threshold = 3.43).

### Correlations between functional brain measures and AHI and MSaO2

**Figure 4:**
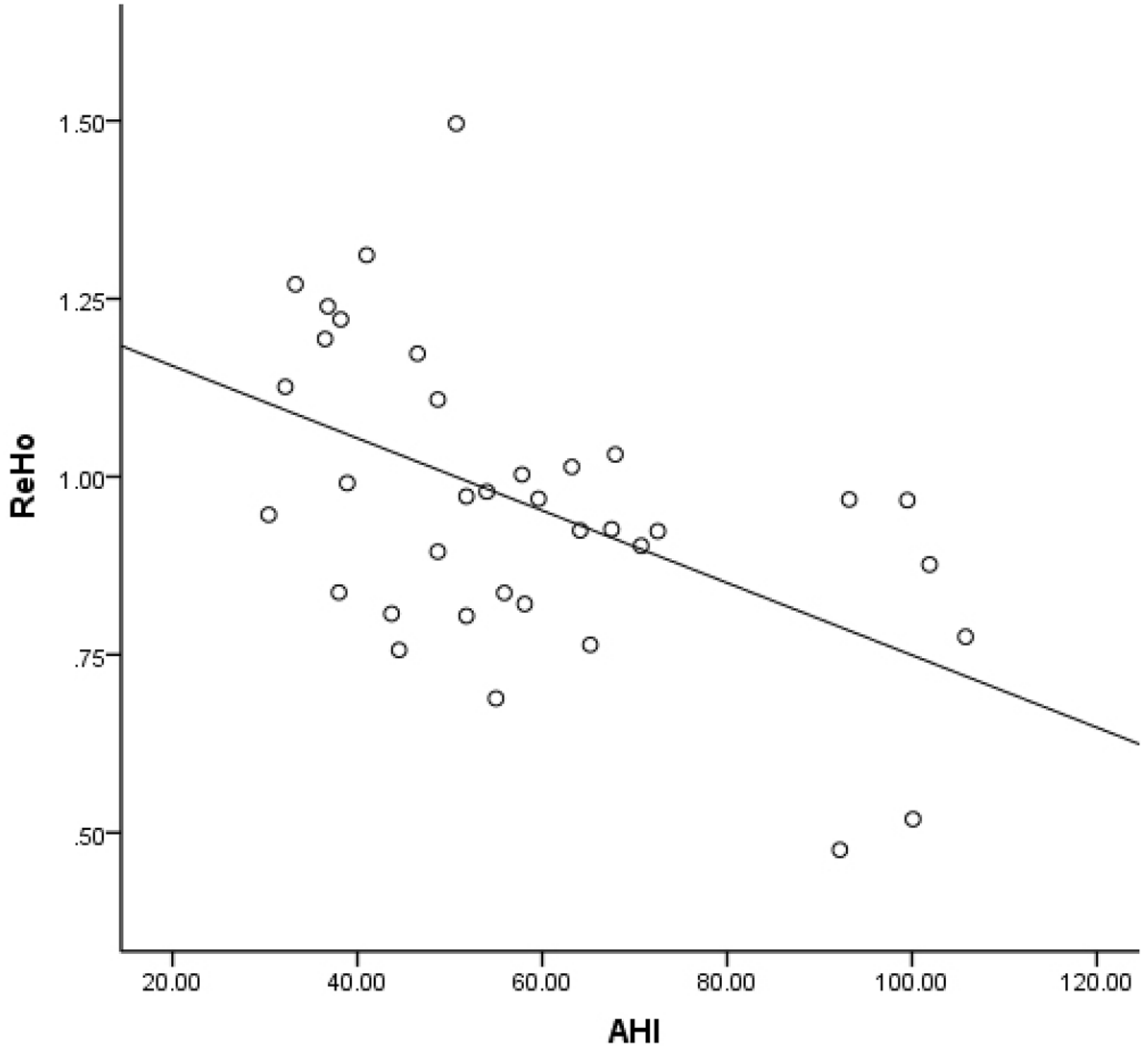
Correlation between brain regions and AHI. In the brain region where ReHo values were higher, ReHo values in the left superior frontal gyrus appear to be negatively related to AHI values (P = 0.001, r = − 0.517). *ReHo* regional homogeneity, *AHI* apnoea-hypopnoea index.

**Figure 5:**
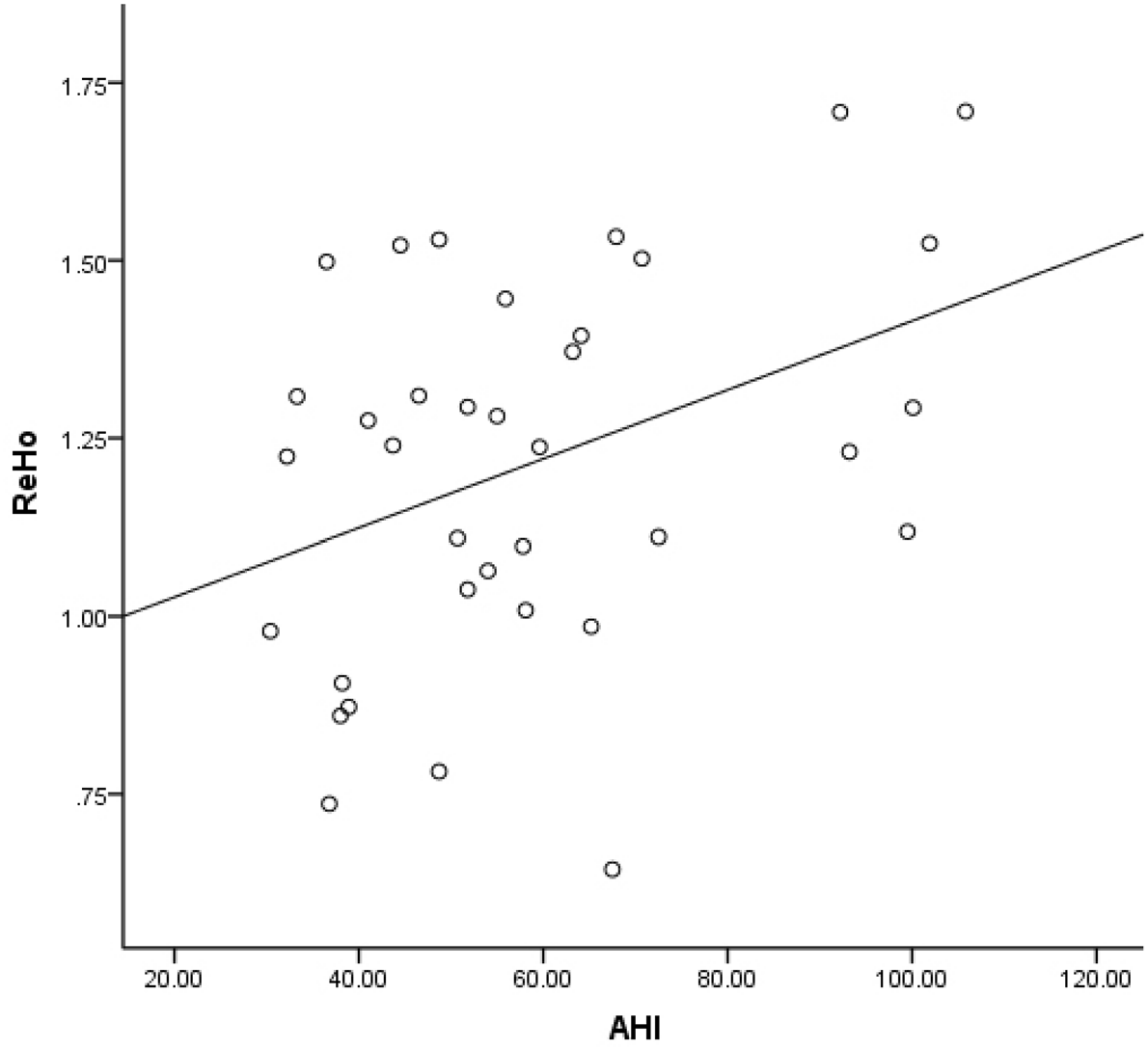
Correlation between brain regions and AHI. In the brain region where ReHo values were higher, ReHo values in the right postcentral gyrus appear to be positively related to AHI values (P = 0.020, r = 0.386). *ReHo* regional homogeneity, *AHI* apnoea-hypopnoea index.

**Figure 6:**
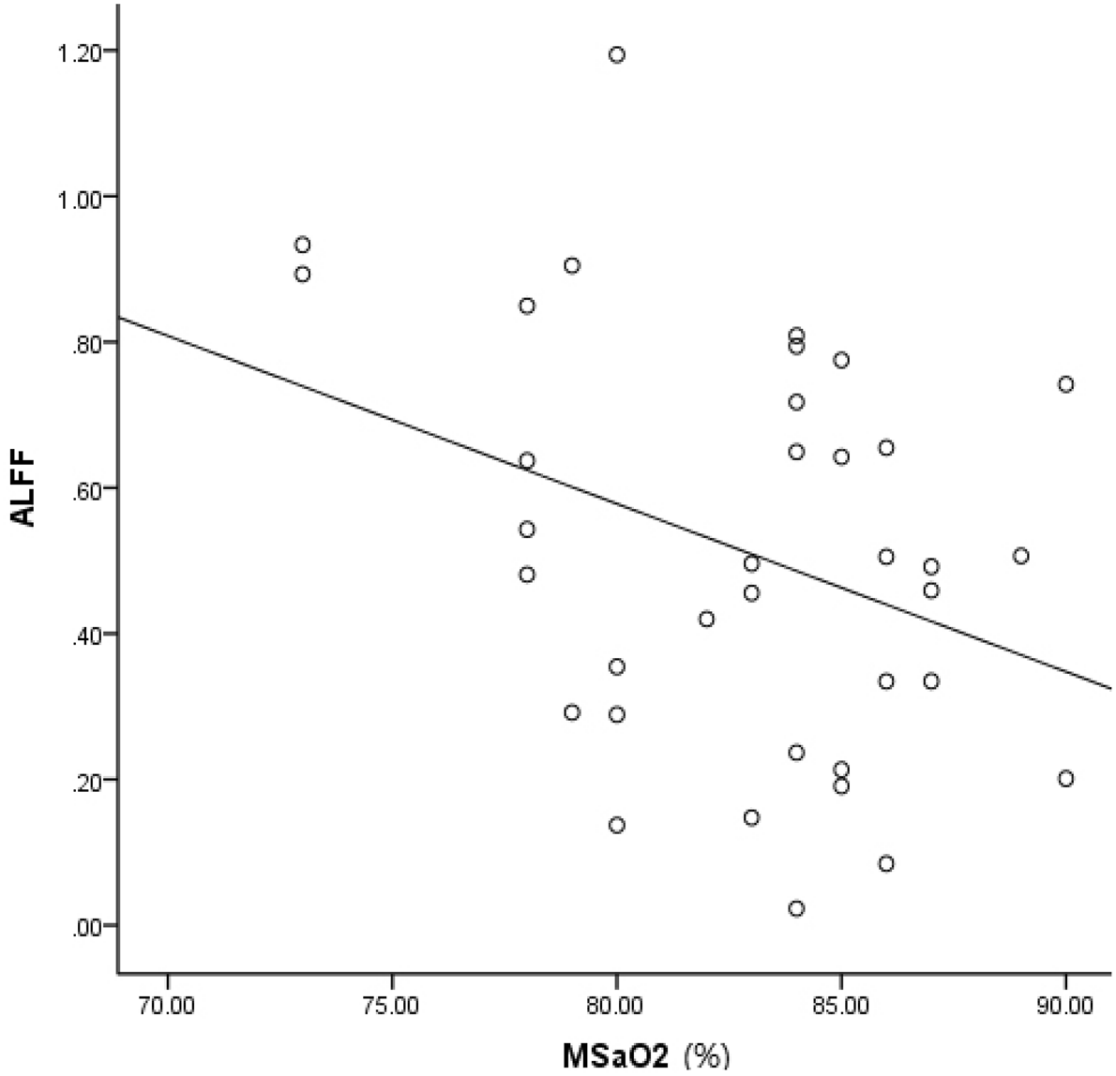
Correlation between brain regions and MSaO2 (%). In the brain region where ALFF values were decreased, ALFF values in the right middle occipital gyrus appear to be negatively related to MSaO2 levels (%) (P = 0.040, r = − 0.344). *ALFF* amplitude of low frequency fluctuation, *MSaO*_*2*_ mean saturation of blood oxygen.

The OSAHS patients and controls had significantly different AHI and MSaO2 levels. In the brain region where ReHo values were higher, ReHo values in the left superior frontal gyrus appear to be negatively related to AHI values (P = 0.001, r = − 0.517, Fig. 4). In another brain region where ReHo values were higher, ReHo values in the right postcentral gyrus appear to be positively related to AHI values (P = 0.020, r = 0.386, Fig. 5). In the brain region where ALFF values were decreased, ALFF values in the right middle occipital gyrus appear to be negatively related to MSaO2 levels (P = 0.040, r = − 0.344, Fig. 6).

## Discussion

In this study, we investigated changes in brain activity in OSAHS patients at high altitude. The current study found that the OSAHS patients had higher ReHo values in the left superior frontal gyrus, right anterior cingulate, left parahippocampus, right postcentral gyrus, right hippocampus, and right precuneus and decreased ReHo values in the left cuneus and left precuneus. The OSAHS patients had higher ALFF values in the right middle cingulate, left medial superior frontal gyrus, right anterior cingulate, right hippocampus and left parahippocampus and decreased ALFF values in the right calcarine, right inferior occipital gyrus, right middle occipital gyrus, left calcarine and right cerebellum_7b. In the OSAHS patients, the PCC showed higher FC with the left caudate and the left thalamus.

As mentioned earlier, BOLD reflects first blood oxygenation and secondly brain functions linked to cellular metabolism. Chronic hypoxia-related changes caused by combination of OSAHS and high altitude and explored by BOLD techniques may be related to:

(1) Anatomical changes due to “long time scale” modifications of the brain connectivity and of local brain functioning, caused by chronic brain oxygenation deprivation, and able to modify the basal activity of the “default mode”.

(2) vascular and metabolic changes due to the “short timescale” adaptation of the vascular functions to hypoxic perturbations, like changes in blood properties (like MSaO_2_), cerebral vasculature adaptation, decreased (or increased by compensation) local metabolism, etc.

For the first case, The Change in brain anatomy may affect the cognitive function of patients. Dosenbach et al.^16^ found that the anterior prefrontal cortex, anterior insula/frontal operculum, basal ganglia and thalamus are part of the cingulo-opercular network (CON), and these regions may be involved in activation, maintenance, and monitoring of task execution. It can be inferred that if a brain region that is part of the CON network is abnormal, it may affect task execution function in OSAHS patients. Surveys conducted by Santarnecchi et al.^17^ indicated that the intrinsic connections between the central anterior gyrus and central posterior gyrus were enhanced in OSAHS patients, and the authors suggested that this enhancement may be related to higher activity of muscle tissue associated with sleep. In the present study, ReHo values in the right postcentral gyrus were higher, indicating that the function of this brain region was changed, which may affect the activity of related muscle tissue and cause sleep disturbance. In this study, brain activity in various regions of the frontal lobe were higher in the OSAHS patients. Thomas et al.^18^ applied a 2-back working memory task to study patients with obstructive sleep-disordered breathing. Their results showed that the dorsolateral prefrontal lobe was always in a negative activation state, and the success rate and response time of patients were not significantly improved after treatment with positive airway pressure. This may explain why working memory has been associated with decreased dorsolateral prefrontal function. Therefore, it can be inferred that the higher brain activity in various regions of the frontal lobe may be related to functional abnormalities such as memory loss. Li et al.^19^ suggested that the activation of the cingulate gyrus was related to the brain network that controls breathing and the function of the autonomic nervous system. Some researchers^20,21^ have found in animal experiments that intermittent hypoxia and sleep fragmentation may lead to neuron loss in the hippocampus, and some researchers^22^ suggested that the hippocampus in OSAHS patients may atrophy, which indicated that the changes in hippocampal brain function in this study may have been due to hypoxia and sleep disorders in patients. The occipital lobe is the visual cortical centre. Some researchers^23^ have suggested that breathing disorders in sleep may promote the occurrence of normal-tension glaucoma (NTG), and the incidences of visual field defects and glaucoma are higher in OSAHS patients.

One of the surprising findings of this study was that in contrast to earlier findings^6,24^, ReHo and ALFF values in the frontal lobe and the precuneus were higher in the OSAHS patients, and the PCC showed higher FC with the left caudate. In addition, we found no regions where functional connectivity with the PCC was significantly decreased. These findings deserve our attention.

Concerning the second case, mentioned field inhomogeneities, which are created by the blood vessel network in the brain, are responsible for the intra- or extra-vascular BOLD contrast^25^. BOLD signals are affected by intra- or extravascular factors. The transformation between paramagnetic deoxyhaemoglobin and diamagnetic oxyhemoglobin affects the magnetic susceptibility of blood, resulting in spatial variation in the water Larmor frequency, which causes an intravascular frequency shift (IFS), ESD is caused by the static magnetic field changes appearing outside large and small vessels. IDA is caused by the changes in blood water T2 in the intravascular compartment. EDD is caused by tissue water diffusing in the internal magnetic field gradients (MFGs), which are created by the vascular system^26^.

The Qinghai-Tibet Plateau is the highest and largest plateau in the world, and it is also known as the "Roof of the World". A series of pathophysiological changes in the human body are caused by the special geographical environment in the plateau area, such as low oxygen pressure, cold temperatures, enhanced ultraviolet radiation and large temperature differences between day and night. High altitude can damage brain blood vessels, and damaged cerebrovascular reactivity can interfere with the transportation of oxygen^27,28^.

Hypoxic hypoxia (decreased partial pressure in dioxygen) is the essence and starting link of pathophysiological changes and may be caused both by high altitude and OSAHS. IFS is related to vessel radii and orientation, so that the vasoconstriction, which is caused by hypoxia at high altitude may decrease the deoxyhaemoglobin fraction in blood vessels and may indirectly affect the BOLD signal. In our study, brain function values of the patient and healthy control groups may have all decreased, but the magnitude of the decrease in values was different, so that the brain function values in some brain regions in the OSAHS patients showed a relatively high state. IDA is related to the content of red blood cells, so erythrocytopenia caused by high altitude may also contribute to changes in brain function measures^26^.

The OSAHS patients and controls had significantly different AHI and MSaO2 levels. In the brain regions where ReHo values were higher, ReHo values in the right postcentral gyrus appeared to be positively related to AHI values, and in the brain region where ALFF values were decreased, ALFF values in the right middle occipital gyrus appeared to be negatively related to MSaO2 levels. These results may indicate that the higher the oxygen content is, the smaller the change in brain function. However, in the brain region where ReHo values were higher, ReHo values in the left superior frontal gyrus appeared to be negatively related to AHI values; the mechanism underlying this complication is currently unknown.

Further exploration of the “short” and “long” timescale determinism of the measure of hypoxic cerebral changes with BOLD may shed light on this aspect.

In conclusion, in high altitude, we found that the brain function of OSAHS patients compared to controls were changed, especially in the right middle cingulate, left medial superior frontal gyrus, right anterior cingulate, right hippocampus and left parahippocampal regions. These changes in brain function in OSAHS patients at high altitude are different from those previously found in OSAHS patients in plains areas. These factors can make patients living at high altitude more aware of the importance of treatment, especially patients at high altitude. To our knowledge, hypoxia can lead to vasoconstriction, and the main reason is the dysfunction of endothelial cells during hypoxia^29^. For patients with OSAHS at high altitude, owing to the more severe hypoxia, the treatment may be of different from that in low altitude areas. In addition to continuous positive airway pressure, cerebral blood flow may be higher by protecting vascular endothelial cells. Some measures to prevent and treat encephalopathy at high altitude may also be applicable to prevent brain changes in OSAHS patients, such as raising the head 30∘ in the supine position^30^.

This research has limitations. Owing to the lack of a reliable cognitive assessment scale, we lack complete clinical information, such as a neuropsychological evaluation. Due to limited quality of non-MRI data, we did not correlate between MRI-derived brain function and EEG or blood, nasal or oral pressure data. However, from the perspective of imaging alone, at least the findings may increase the attention of OSAHS patients at high altitude. Further studies on this basis requires also to expand the number of samples to increase the statistical power of our results.

## Conclusions

The changes in some brain functions in OSAHS patients at high altitude are different from those in plains areas. These may be due to the high altitude hypoxia environment and attention should be paid to monitor OSAHS patients at high altitude, owing to potentially unpredictable and more severe neurological and sleep changes than in plain areas. Further studies focused on the effect of altitude in OSAHS should explore the consequences specific functional changes described in this study.

## Methods

### Subjects

Thirty-six males from high altitude regions (2,000–3,000 m) who were diagnosed with OSAHS at Qinghai University Affiliated Hospital were enrolled in this study. Considering that hypertension may have an impact on brain function, we collected patients with blood pressure below 130/80 mm/Hg. The patient inclusion criteria were as follows: Han Chinese individuals (different ethnic groups, such as Tibetans, have different lifestyles and may have different adaptability to anoxic environments. Therefore, they may have different brain structures and functional impairments resulting from OSAHS^31^); newly diagnosed patients without any treatment prior to diagnosis; individuals with no serious cardiovascular or cerebrovascular diseases (e.g., severe myocardial infarction, arrhythmia, stroke); right-handed individuals. The patient exclusion criteria were as following: sleep disorder other than OSAHS; mental or neurological disease; alcohol or history of taking psychotropic substances; and magnetic resonance imaging contraindications. The control group recruited 38 healthy male volunteers who matched the OSAHS group on altitude, age, and education level. The subjects in the control group were all right-handed and Han Chinese. In the control group, subjects had no intracranial disease, physical examination results were normal as screened by professional physicians, no clinical symptoms of OSAHS, and the normal indicators reported through polysomnography (PSG) monitoring. The altitude of the city where the scanner was located is 2,295 m. All patients and healthy controls come from high altitude areas (2000–3,000 m) and are native.

### PSG

PSG (Alice 6, Philips, USA) was performed in all participants. To ensure the accuracy of monitoring results, the subjects were asked to avoid issues affecting sleep monitoring before PSG. Subjects should remain awake and maintain stable emotions before the examination. Coffee, tea, alcohol and sedative and hypnotic drugs were prohibited before examination. Bathing, shampooing and shaving before examination helped make the electrodes contact the skin well. PSG included synchronous detection of electroencephalogram (EEG), electrooculogram (EOG), electromyogram (EMG), electrocardiogram (ECG), mean saturation of blood oxygen (MSaO2), oral and nasal pressure and heat sensitive airflow, thoracic and abdomen movements, body movements, leg movements, snoring, etc. The subjects were monitored with the Alice 6 model for more than 7 h. OSAHS was diagnosed according to the American Academy of Sleep Medicine (AASM, 2007) guidelines.

### Image acquisition

Resting-state fMRI images were collected on a Philips 3.0T MR scanner equipped with a standard 8-channel head coil. The subjects were instructed to be awake during the entire rs-fMRI scan. First, DWI and conventional MRI sequences were performed in all subjects to remove subjects with brain abnormalities. For the three-dimensional T1-weighted images (3D-T1), the ultrafast field echo sequence (TFE) parameters were as follows: repetition time, 7.5 ms; echo time, 3.7 ms; flip angle, 7 degrees; slice thickness, 2 mm; and matrix size, 256 × 256. Finally, 176 images were obtained. For the rs-fMRI scan, the echo planar imaging (EPI) sequence parameters were as follows: repetition time, 2,500 ms; echo time, 30 ms; flip angle, 90 degrees; slice thickness, 3.5 mm; slice gap, 0.35 mm; FOV, 224 mm × 224 mm. Finally, 5,250 images were obtained.

### ReHo and ALFF analysis

Acquired images were separated and processed to obtain resting-state series. Data were processed by SPM8 (Statistical Parametric Mapping) software (https://www.fil.ion.ucl.ac.uk./spm8/) based on the MATLAB 2010 b (MathWorks, Natick, Massachusetts) platform. The first 10 time points in the rs-fMRI series were discarded, and we preserved the data from 140 time points in the time series. Data with head motion greater than 1.5 mm in x, y, z directions or 1.5 degrees were excluded. The data for each individual were normalized to Montreal Neurological Institute (MNI) space. Images were resampled to 3 mm^3^ resolution and smoothed with a 8 mm^3^ full-width at half-maximum (FWHM) Gaussian kernel. The filter (0.01 < f < 0.08 Hz) was used to reduce the effects of low frequency drift and high frequency physiological noise. The preprocessed data were de-linearly drifted and filtered using the ALFF tool in REST 1.8 software (https://www.restfmri.net/forum/REST_V1.8). ALFF values were obtained after the power spectrum of the signal at 0.01 to 0.08 Hz was squared. After normalization, standardized ALFF values were obtained. The Resting-State fMRI Data Analysis Toolkit (https://www.restfmri.net) was used to obtain Kendall’s coefficient concordance (KCC), which can reflect synchronous activity in the brain. ReHo values for each subject were obtained by evaluating the KCC between each voxel in the brain and the nearest 26 voxel time series around it^13^. The computing formula is

$${\text{W}}\, = \,(\sum \left( {{\text{R}}_{{\text{i}}} } \right)^{{\text{2}}} - {\text{n}}(\sum {\text{R}})^{{\text{2}}} )\, \times \,{\text{12k}}^{{\text{2}}} ({\text{n}}^{{\text{3}}} - {\text{n}}).$$

In this formula, W is the KCC among the given voxels and ranged from 0 to 1. Ri refers to the sum rank of the ith time point. R = [(n + 1)K]/2 means the average value of Ri. n refers to the number of ranks; here, n = 140. K refers to the number of time series within a measured cluster; here, K = 27.

### FC analysis

The preprocessed data were de-linearly drifted by REST software, and bandpass filtering (0.01–0.08 Hz) was performed for each time series. Generally, the seed point correlation analysis method selects the PCC as the seed point^32^. Then, we analysed the correlations between the PCC and the whole brain voxel time series, calculated the correlation coefficient r, and converted r into a Z value conforming to the normal distribution by Fisher Z transformation. Finally, a single-sample t-test was performed on the OSAHS group and the control group using REST software, and then a two-sample t-test was performed. REST software was used to render functionally connected abnormal brain regions. All results were corrected using AlphaSim for multiple comparisons. The pixels that meet the statistical threshold were projected into the Montreal standard template space, and X, Y, and Z represented their three-dimensional coordinates.

### Statistical analysis

Statistical analysis tools in REST were used for the ReHo, ALFF and FC data. Two sample t-tests were conducted. Statistical parameters for ReHo analyses: P < 0.001 (cluster size > 22, AlphaSim correction); statistical parameters for ALFF analyses: P < 0.001 (cluster size > 22, AlphaSim correction). Statistical parameters for FC analyses: in the one-sample T test, P < 0.001 (cluster size > 22, AlphaSim correction); in the two-sample t-test, P < 0.001 (cluster size > 22, AlphaSim correction). Statistical analyses of the clinical data were performed using two independent sample T-tests with SPSS software (version 19.0). Correlation analyses were used between brain areas with altered brain function and AHI, BMI and MSaO2 levels in the patient group. Pearson correlations were used to evaluate the relationship between brain areas with altered brain function and AHI, BMI and MSaO2 levels in the patient group. The statistical results were considered statistically significant at P < 0.05.

### Ethical approval

All procedures performed in studies involving human participants were in accordance with the ethical standards of the institutional and/or national research committee and with the 1964 Helsinki declaration and its later amendments or comparable ethical standards. All subjects signed informed consent, and all experimental protocols were approved by the ethics committee of the Affiliated Hospital of Qinghai University.
